# Cardiovascular Variability Analysis and Baroreflex Estimation in Patients with Type 2 Diabetes in Absence of Any Manifest Neuropathy

**DOI:** 10.1371/journal.pone.0148903

**Published:** 2016-03-17

**Authors:** Sílvia Cristina Garcia de Moura-Tonello, Alberto Porta, Andrea Marchi, Alessandra de Almeida Fagundes, Cristina de Oliveira Francisco, Patrícia Rehder-Santos, Juliana Cristina Milan-Mattos, Rodrigo Polaquini Simões, Mariana de Oliveira Gois, Aparecida Maria Catai

**Affiliations:** 1 Department of Physical Therapy, Federal University of São Carlos, São Carlos, Brazil; 2 Department of Biomedical Sciences for Health, University of Milan, Milan, Italy; 3 Department of cardiothoracic, Vascular Anesthesia and Intensive Care, IRCCS Policlinico San Donato, Milan, Italy; 4 Department of Electronics Information and Bioengineering, Politecnico di Milano, Milan, Italy; Niigata University Graduate School of Medical and Dental Sciences, JAPAN

## Abstract

**Introduction:**

Indexes derived from spontaneous heart period (HP) and systolic arterial pressure (SAP) fluctuations can detect autonomic dysfunction in individuals with type 2 diabetes mellitus (DM) associated to cardiovascular autonomic neuropathy (CAN) or other neuropathies. It is unknown whether HP and SAP variability indexes are sensitive enough to detect the autonomic dysfunction in DM patients without CAN and other neuropathies.

**Methods:**

We evaluated 68 males aged between 40 and 65 years. The group was composed by DM type 2 DM with no manifest neuropathy (n = 34) and healthy (H) subjects (n = 34). The protocol consisted of 15 minutes of recording of HP and SAP variabilities at rest in supine position (REST) and after active standing (STAND). The HP power in the high frequency band (HF, from 0.15 to 0.5 Hz), the SAP power in the low frequency band (LF, from 0.04 to 0.15 Hz) and BRS estimated via spectral approach and sequence method were computed.

**Results:**

The HF power of HP was lower in DM patients than in H subjects, while the two groups exhibited comparable HF power of HP during STAND. The LF power of SAP was similar in DM and H groups at REST and increased during STAND in both groups. BRSs estimated in the HF band and via baroreflex sequence method were lower in DM than in H and they decreased further during STAND in both populations.

**Conclusion:**

Results suggest that vagal control of heart rate and cardiac baroreflex control was impaired in type 2 DM, while sympathetic control directed to vessels, sympathetic and baroreflex response to STAND were preserved. Cardiovascular variability indexes are sensitive enough to typify the early, peculiar signs of autonomic dysfunction in type-2 DM patients well before CAN becomes manifest.

## Introduction

Type 2 diabetes mellitus (DM) has increased its prevalence worldwide and it has been considered as a public health problem due to its huge impact on the life quality and expectancy of the individual and on the community in relation to the high cost of its control, management and treatment [1,2]. When type 2 DM is associated to cardiovascular autonomic neuropathy (CAN), an autonomic dysfunction is expected and usually detected [3–8]. However, it is unknown whether the autonomic dysfunction should be considered a consequence of the manifestation of neuropathy or a primary effect of type 2 DM and, if an autonomic dysfunction can be exclusively linked to type 2 DM, it can be detected via the analysis of the spontaneous fluctuations of heart period (HP) and systolic arterial pressure (SAP) [9].

The early diagnosis of the autonomic dysfunction is very important and it must be done preferably before CAN become manifest to favor its early management and prevention of serious consequences including the risk of cardiovascular morbidity and mortality [10]. Some studies showed that indexes derived from spontaneous fluctuations of HP and SAP, including univariate and bivariate markers, have a higher sensitivity and specificity than conventional autonomic function tests in detecting the autonomic dysfunction in DM patients [3–5]. Indeed, spectral HP and SAP indexes and BRS estimates were found to be significantly modified in DM individuals [3,6–8].

In this context, studies evaluating the autonomic function in individuals with type 2 DM without CAN and asymptomatic for any other type of neuropathy might be important to clarify the primary effect of type 2 DM on the cardiovascular control and facilitate the early management of this pathology. Therefore, we hypothesized that autonomic indexes derived from SAP and HP spontaneous fluctuations in individuals with type 2 DM without CAN and asymptomatic for any other type of neuropathy can indicate the early impairment of the cardiac control.

According to the hypothesis we computed spectral HP and SAP variability indexes and BRS estimates [11–13] in individuals with type 2 DM without CAN and asymptomatic for any other type of neuropathy at rest in supine position (REST) and during active standing (STAND). STAND was exploited to probe the ability of the cardiovascular control to cope with a typical stressor of the cardiovascular system (i.e. the reduction of the venous return in relation to the modification of posture) via a vagal inhibition and sympathetic activation [14–17].

## Materials and Methods

### Population

We evaluated 68 males, aged between 40 and 65 years (Table 1). Table 2 reported the medications relevant to the considered population. The group was composed by patients with DM without neuropathy (n = 34) and healthy (H) subjects (n = 34). They were all non-smokers and non-habitual drinkers. Additional exclusion criteria were as follows: ECG with alterations, and/or myocardial ischemia and/or cardiovascular pathologies, abnormalities in the respiratory, neurological, and osteomyoarticular systems, use of illicit drugs or any medications known to interfere with cardiovascular control and diagnosis of any manifest neuropathy. The following standard criteria were used to exclude from our DM patients those with CAN: resting heart rate larger than 100 beats per minute, abnormal value of slow deep breathing autonomic test, anomalous value of the 30:15 ratio of heart rate in response to STAND, abnormal heart rate response to the Valsalva maneuver (i.e. the typical ratio of the longest HP to the shortest one is usually larger than 1.2), atypical orthostatic hypotension in reaction to STAND (the normal response is a fall of SAP less than 10 mmHg) [18,19]. The absence of peripheral neuropathy and other type of neuropathies was checked by evaluating the sensitivity to 10.0 g Semmes-Weinstein monofilament and by clinical anamnesis [20].

**Table 1:** Characteristics of population.

Characteristics	H	DM
Age (years)	54.50 ± 5.96	54.0 ± 6.05
Body mass (Kg)	78.97 ± 12.35	82.08 ± 13.29
Height (m)	1.72 ± 0.07	1.72 ± 0.08
BMI (Kg/m^2^)	26.74 ± 3.49	27.92 ± 3.93
Time of diabetes (years)	-	10.99 ± 7.65
HR (bpm)	65.41 ± 9.96	69.74 ± 8,69*
SAP (mmHg)	120.5 (111–126)	131.5 (125–146)*
DAP (mmHg)	68.74 ± 6.37	74.15 ± 8,83*
Laboratory Exams		
CRP (mg/dL)	0.84 (0.54–1.96)	1.08 (0.41–2.48)
HbA1c (%)	5.5 (5.3–5.7)	7.7 (6.8–8.7)*
Fasting plasma glucose (mg/dL)	112.6 (96.9–145.5)	174.2 (119.7–343)*
Insulin	5.85 (4.25–7.55)	10.75 (6.40–14.40)*
Total Cholesterol (mg/dL)	204 ± 37.46	209 ± 43.09
HDL-Cholesterol (mg/dL)	48.0 (39.0–52.5)	42.0 (36.0–48.0)
LDL-Cholesterol (mg/dL)	128.16 ± 32.29	127.39 ± 41.83
VLDL-Cholesterol (mg/dL)	23.0 (18.25–31.75)	36.0 (23.75–43.00)
Triglycerides (mg/dL)	119 (92.5–166.5)	184 (119.0–216.0)*
Risk factors		
Hypertension	-	15 (44.12%)

H = group with healthy subjects; DM = group with type 2 diabetes without neuropathy; BMI = body mass index; CRP = C-reactive protein; HbA1c = glycated hemoglobin; HR = heart rate at rest in supine position; SAP = systolic arterial pressure at rest in supine position; DAP = diastolic arterial pressure at rest in supine position. The data are presented in mean ± standard deviation or medians and first-to-third quartile range in parentheses.

The symbol * indicates a *p* < 0.05 (H vs DM).

**Table 2:** Medications in the considered population.

Medications	H	DM
Diabetes medications	-	34 (100%)
Metformin	-	11 (32.35%)
DPP-4 inhibitor	-	1 (2.94%)
Insulin	-	3 (8.82%)
Metformin + Sulfonylureas	-	10 (29.41%)
Metformin + DPP-4 inhibitor	-	2 (5.88%)
Metformin + Sulfonylureas + DPP-4 inhibitor	-	1 (2.94%)
Metformin + Insulin	-	6 (17.65%)
Antihypertensive drugs	-	15 (44.12%)
ACE inhibitor	-	3 (8.82%)
Calcium channel blocker (amlodipina)	-	1 (2.94%)
Angiotensin II receptor antagonist	-	6 (17.65%)
Hydrochlorothiazide	-	1 (2.94%)
Hydrochlorothiazide + ACE inhibitor	-	2 (5.88%)
Hydrochlorothiazide + Angiotensin II receptor antagonist	-	2 (5.88%)
Hypolipidemic drug	-	9 (14.71%)

H = group with healthy subjects; DM = group with type 2 diabetes without neuropathy; ACE = angiotensin converting enzyme; DPP-4 = Dipeptidyl peptidase-4. The data are presented as number of subjects and percentage between rounded parentheses.

This study was carried out in according to the Declaration of Helsinki for medical research involving humans. All subjects were informed about the experimental procedures, read and signed an informed consent form. This study was approved by the Human Research Ethics Committee of the Federal University of São Carlos, Brazil (n. 35068814.2.0000.5504).

### Experimental protocol

The volunteers were instructed to avoid consuming food and/or drinking stimulating or alcoholic beverages as well as practicing moderate or heavy exercise within 24 hours before the evaluation. All the experimental procedures were carried out in the morning in temperature-controlled room (22–23°C) with a relative air humidity of 50–60%, at the Cardiovascular Physiotherapy Laboratory at the Federal University of São Carlos, São Carlos, Brazil. Only blood collection was performed at the Clinical Analysis Laboratory. The subjects were first interviewed and examined to verify if they had a regular night sleep and if they were in good health. Then, blood collection was made before starting the experimental procedures. Half one hour was allowed between the blood collection and the beginning of the protocol. During this period the subject consumed a light breakfast in the laboratory. Before starting the protocol, all volunteers were familiarized with equipment and facilities of the laboratory. The subjects were instructed to lie in the supine position, breath spontaneously, and avoid moving and/or talking during the experiment. The ECG from lead II was recorded (BioAmp Power Lab, ADInstruments, Australia) together with continuous plethysmographic arterial pressure from the middle finger of left hand (Finometer PRO, Finapress Medical System, The Netherlands) and respiratory movements by thoracic belt (Marazza, Monza, Italy). All signals were simultaneously digitalized with a sampling rate of 1 KHz (Power Lab, ADInstruments, Australia). All subjects remained at REST for 15 minutes to stabilize the cardiovascular parameters before starting the recording. The arterial pressure signal was cross-calibrated using a measure provided by a sphygmomanometer at the onset of the REST. The auto-calibration procedure of the arterial pressure device was switched off after the first automatic calibration at the onset of the session. The recording at REST lasted 15 minutes. After this, the volunteers were instructed to modify their posture to STAND and remain in this position from 15 minutes.

### Extraction of the beat-to-beat variability series

After detecting the R-wave peak on the ECG, its apex was located using a parabolic interpolation. The temporal distance between two consecutive R-wave apexes was computed and utilized as an approximation of HP [21]. The maximum of arterial pressure (AP) inside HP was defined as SAP, and the *i*-th SAP [i.e., SAP(*i*)] was taken inside the *i*-th HP [i.e., HP(*i*)], where *i* is the cardiac beat counter.

The occurrences of the R-wave and SAP peaks were carefully checked to avoid erroneous detections or missed beats. If isolated ectopic beats affected HP and SAP values, these measures were linearly interpolated using the closest values unaffected by ectopic beats. HP = {HP(*i*), *i* = 1,…,*N*} and SAP = {SAP(*i*), *i* = 1,…, *N*} were extracted on a beat-to-beat basis, where *N* is the series length. Synchronous sequences of *N* = 256 consecutive HP and SAP measures were selected in a random position inside the REST and STAND periods. The length of the series was chosen to fulfill the requirements of short-term cardiovascular variability analysis [9] and to speed up fast Fourier transform exploited to build the surrogate set utilized to test the significance of the HP-SAP association. The series were linearly detrended. If evident nonstationarities, such as very slow drifting of the mean or sudden changes of the variance, were visible despite the linear detrending, the random selection was carried out again. The stationarity of mean and variance of the selected sequences was finally tested according to [22]. Analysis during STAND was performed after five minutes from the onset of the maneuver to facilitate the detection of stationary sequences. The mean of HP and SAP, μ_HP_ and μ_SAP_, and the variance of HP and SAP, σ^2^_HP_ and σ^2^_SAP_, were computed and expressed as ms, mmHg, ms^2^ and mmHg^2^.

### Power spectral analysis

The power spectrum was estimated according to a univariate parametric approach fitting the series according to an autoregressive model [21]. The autoregressive spectral density was factorized into components, each of them characterized by a central frequency. A spectral component was labeled as low frequency (LF, from 0.04 to 0.15 Hz) or high frequency (HF, from 0.15 to 0.4 Hz) if its central frequency belonged to the LF or HF band. The LF and HF powers were defined as the sum of the powers of all LF and HF spectral components, respectively [9]. The HF power of HP series was expressed in absolute units (ms^2^), indicated as HF_HP._ The HF_HP_ power reflects vagal modulation directed to the heart [23], whereas the LF power of SAP series, expressed in absolute units (mmHg^2^) and indicated as LF_SAP_, reflects efferent sympathetic modulation directed to vessels [11]. The LF and HF bands of HP and SAP series was calculated and utilized to compute the BRS in the frequency domain.

### Frequency domain BRS assessment

The computation of frequency domain BRS was based on spectral approach [12]. The BRS was calculated as the square root of the ratio of LF_HP_ to LF_SAP_ power, indicated as α_LF_, and as the square root of the ratio of HF_HP_ to HF_SAP_ power, indicated as α_HF_ [24]. The BRS indexes were expressed in ms/mmHg. To be reliably estimated α_LF_ and α_HF_ HP and SAP variabilities must be significantly associated with HP fluctuations lagging behind SAP variations [25,26]. The fulfillment of this condition was tested according to the calculation of squared coherence (K^2^_HP-SAP_) and phase spectrum (Ph_HP-SAP_). K^2^_HP-SAP_ was computed as the ratio of the square HP-SAP cross-spectrum modulus divided by the product of the power spectra of HP and SAP series, while Ph_HP-SAP_ was the phase of the HP-SAP cross-spectrum [27]. K^2^_HP-SAP,_ expressed in dimensionless units, ranged from 0 to 1 indicating a perfect uncorrelation and a full correlation respectively. Ph_HP-SAP_, expressed in radians, ranged between +π and –π radians indicating both phase opposition. K^2^_HP-SAP_ and Ph_HP-SAP_ were sampled in correspondence of the weighted average of the central frequencies of the LF and HF components found in the SAP series (the weights were the powers of the components) and indicated as K^2^_HP-SAP_(LF), K^2^_HP-SAP_(HF), Ph_HP-SAP_(LF) and Ph_HP-SAP_(HF) respectively. We checked that at the frequency of interest K^2^_HP-SAP_ was larger than a threshold computed according to a set of 100 isospectral isodistributed uncoupled SAP and HP surrogates [27] and Ph_HP-SAP_ was negative, thus assuring that HP and SAP series were significantly coupled and HP fluctuations lagged behind SAP changes.

### Time domain BRS assessment

The time domain of BRS was based on sequence method [13] as implemented in [24]. The baroreflex sequence technique relies on the scanning of the HP and SAP series in the search for sequences with length of four consecutive beats characterized by the contemporaneous increase (up sequence) or decrease (down sequence) of HP and SAP and referred to as baroreflex sequences. Only sequences with the following features were labeled as baroreflex sequences: 1) the total HP variation was larger than 5 ms; 2) the total SAP variation was larger than 1 mmHg; and 3) the correlation coefficient in the plane [SAP(*i*),HP(*i*)] was larger than 0.85. The slope of the regression line in the plane [SAP(*i*),HP(*i*)] was calculated and averaged over all BRS sequences and indicated as α_SEQ_. BRS was also calculated by separating the up sequences from the down ones.

### Statistical analysis

Normality of the data distribution was verified by the Kolmogorov-Smirnov test. The unpaired t test, or Mann-Whitney test when applicable, was used to compare characteristics of the population and results of the laboratory exams. Two ways analysis of variance was utilized to compare univariate and bivariate time and frequency domain indexes between groups within the same experimental condition and between experimental conditions within the same group (Holm-Sidak test for multiple comparisons, one factor repetition). The data were analyzed using a commercial statistical program (Sigmaplot, ver.11.0, Systat Software, San Jose, CA, USA). A *p*<0.05 was always considered to be significant.

## Results

Table 1 shows age, anthropometric characteristics, heart rate, SAP and diastolic arterial pressure, results of the laboratory exams and risk factors. Table 2 reports medications of the DM population. H and DM groups were different in relation to glycated hemoglobin (HbA1c), fasting plasma glucose, insulin and triglycerides. Heart rate, SAP and diastolic arterial pressure were higher in DM patients than in H subjects.

### Time and frequency domain HP and SAP parameters

**Fig 1:**
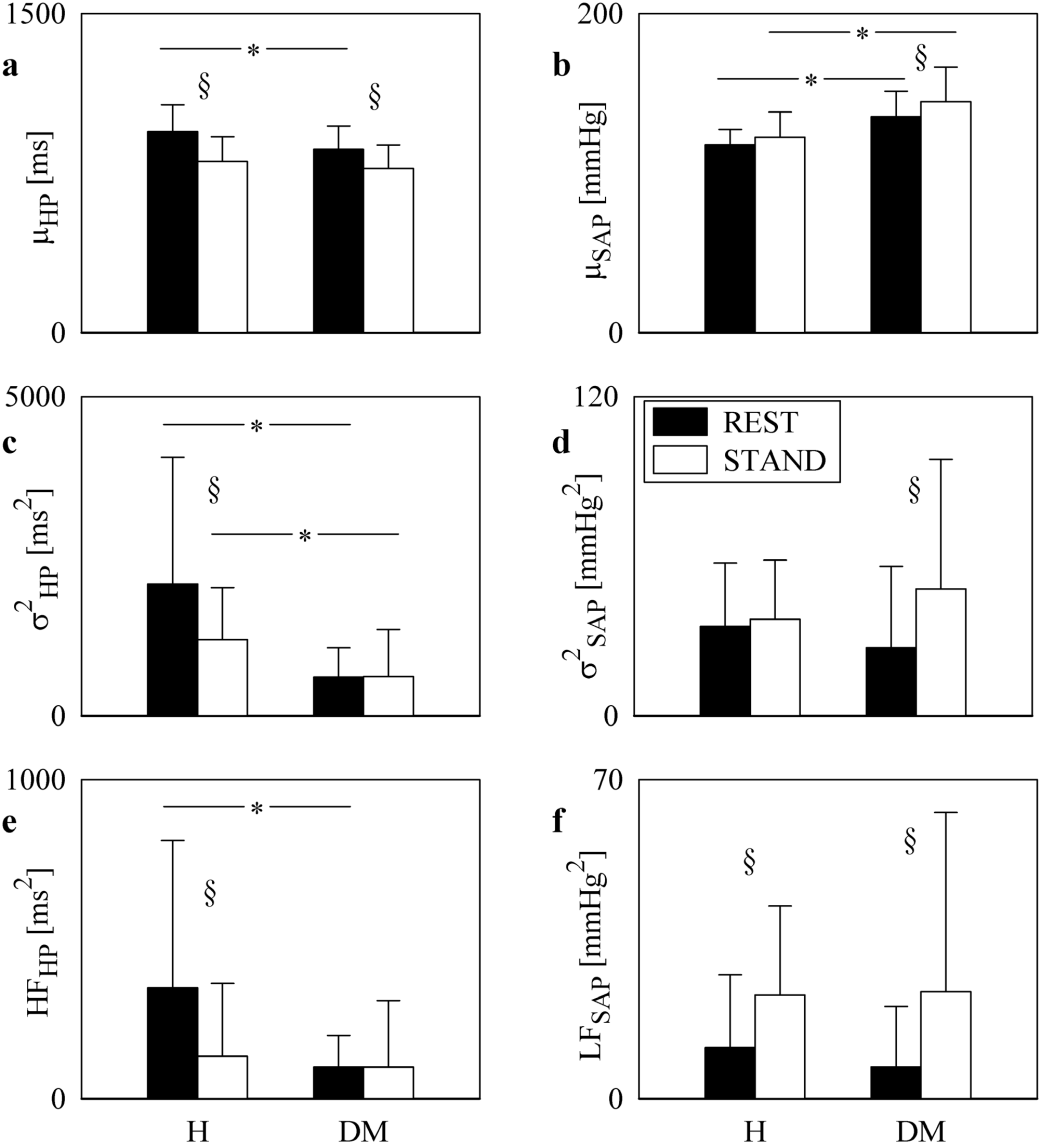
Univariate time and frequency domain analyses of HP and SAP series. The grouped bargraphs show the results of time and frequency domain analyses of HP and SAP series as a function of the group (i.e. H and DM individuals) at REST (black bars) and during STAND (white bars). The graphs are relevant to μ_HP_ (a), μ_SAP_ (b), σ^2^_HP_ (c), σ^2^_SAP_ (d), HF_HP_ (e), and LF_SAP_ (f). Values are given as mean plus standard deviation. The symbol * indicates differences between groups (i.e. H vs DM) with *p*<0.05, while the symbol § indicates differences between experimental conditions (i.e. REST vs STAND) with *p*<0.05.

The grouped bargraphs of Fig 1 show the results of time and frequency domain analyses of HP and SAP series as a function of the group (i.e. H and DM individuals) at REST (black bars) and during STAND (white bars). Values are given as mean plus standard deviation. The μ_HP_ decreased significantly during STAND in both groups. DM patients had μ_HP_ lower than H subjects at REST, but the two groups were indistinguishable during STAND (Fig 1a). μ_SAP_ was significantly larger in the DM group than in H subjects both at REST and during STAND, but STAND affected only the DM group (Fig 1b). The σ^2^_HP_ was significantly reduced in DM group both at REST and during STAND but the effect of STAND was visible only in the H group (Fig 1c). The σ^2^_SAP_ did not distinguish the groups within the same experimental condition (Fig 1d). While STAND increased σ^2^_SAP_ in DM subjects, STAND did not influence σ^2^_SAP_ in H subjects (Fig 1d). The HF_HP_ power was significantly smaller in DM patients than in H individuals at REST, while the values of the HF_HP_ power were similar in the two populations during STAND (Fig 1e). The decrease of the HF_HP_ power during STAND was remarkable in H subjects, while it was negligible in DM patients (Fig 1e). LF_SAP_ power did not separate the two groups but the effect of STAND increasing the LF_SAP_ power was visible in both H and DM individuals (Fig 1f).

### HP-SAP squared coherence and phase analyses

**Fig 2:**
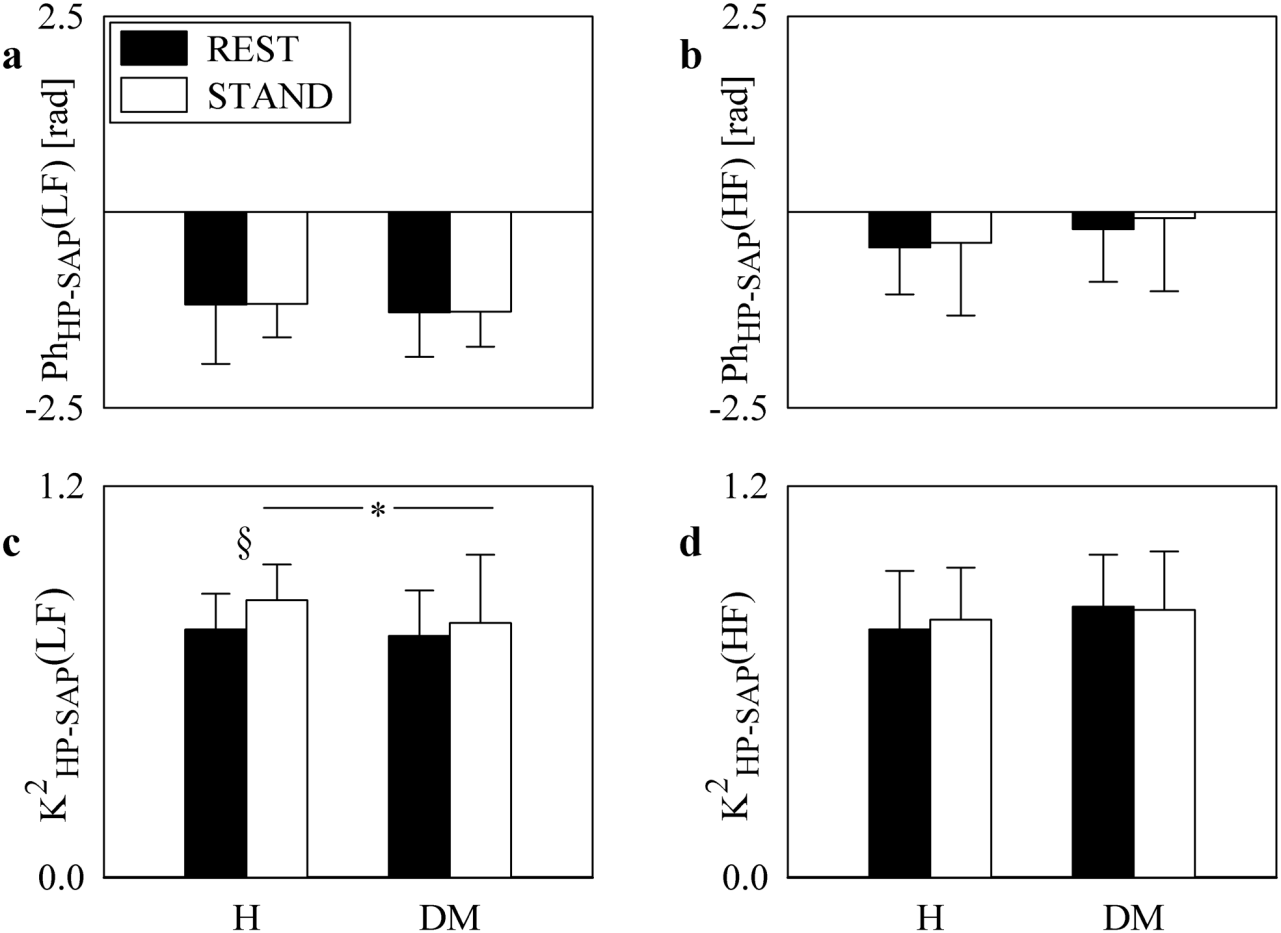
HP-SAP squared coherence and phase analyses. The grouped bargraphs show the results of phase, Ph_HP-SAP_, and squared coherence, K^2^_HP-SAP_, analyses between HP and SAP series as a function of the group (i.e. H and DM individuals) at REST (black bars) and during STAND (white bars). The graphs are relevant to Ph_HP-SAP_(LF) (a), Ph_HP-SAP_(HF) (b), K^2^_HP-SAP_(LF) (c), and K^2^_HP-SAP_(HF) (d). Values are given as mean plus standard deviation. The symbol * indicates differences between groups (i.e. H vs DM) with *p*<0.05, while the symbol § indicates differences between experimental conditions (i.e. REST vs STAND) with *p*<0.05.

The grouped bargraphs of Fig 2 show the results of phase (Fig 2a and 2b) and squared coherence (Fig 2c and 2d) analyses between HP and SAP series as a function of the group (i.e. H and DM individuals) at REST (black bars) and during STAND (white bars). Values are given as mean plus standard deviation. While Ph_HP-SAP_(LF) is significantly smaller than 0 regardless of the experimental condition (i.e. REST or STAND) and group (i.e. H or DM) (Fig 2a), Ph_HP-SAP_(HF) exhibited both positive and negative values (Fig 2b). Neither experimental condition nor group affected Ph_HP-SAP_(LF) and Ph_HP-SAP_(HF) (Fig 2a and 2b). While K^2^_HP-SAP_(HF) was not influenced by experimental condition or group (Fig 2d), K^2^_HP-SAP_(LF) increased during STAND compared to REST in H group and it was significantly smaller in DM group than in H subjects during STAND (Fig 2c). The prerequisite of significant K^2^_HP-SAP_ (i.e. larger than the threshold set with surrogate analysis) and Ph_HP-SAP_ smaller than 0 was fulfilled in the LF band in more than 90% regardless of group and experimental condition. The same prerequisite was fulfilled in the HF band in 73.53% of H subjects and 67.65% of DM patients at REST and in 58.82% of H subjects and 55.88% of DM patients during STAND.

### Assessment of BRS

**Fig 3:**
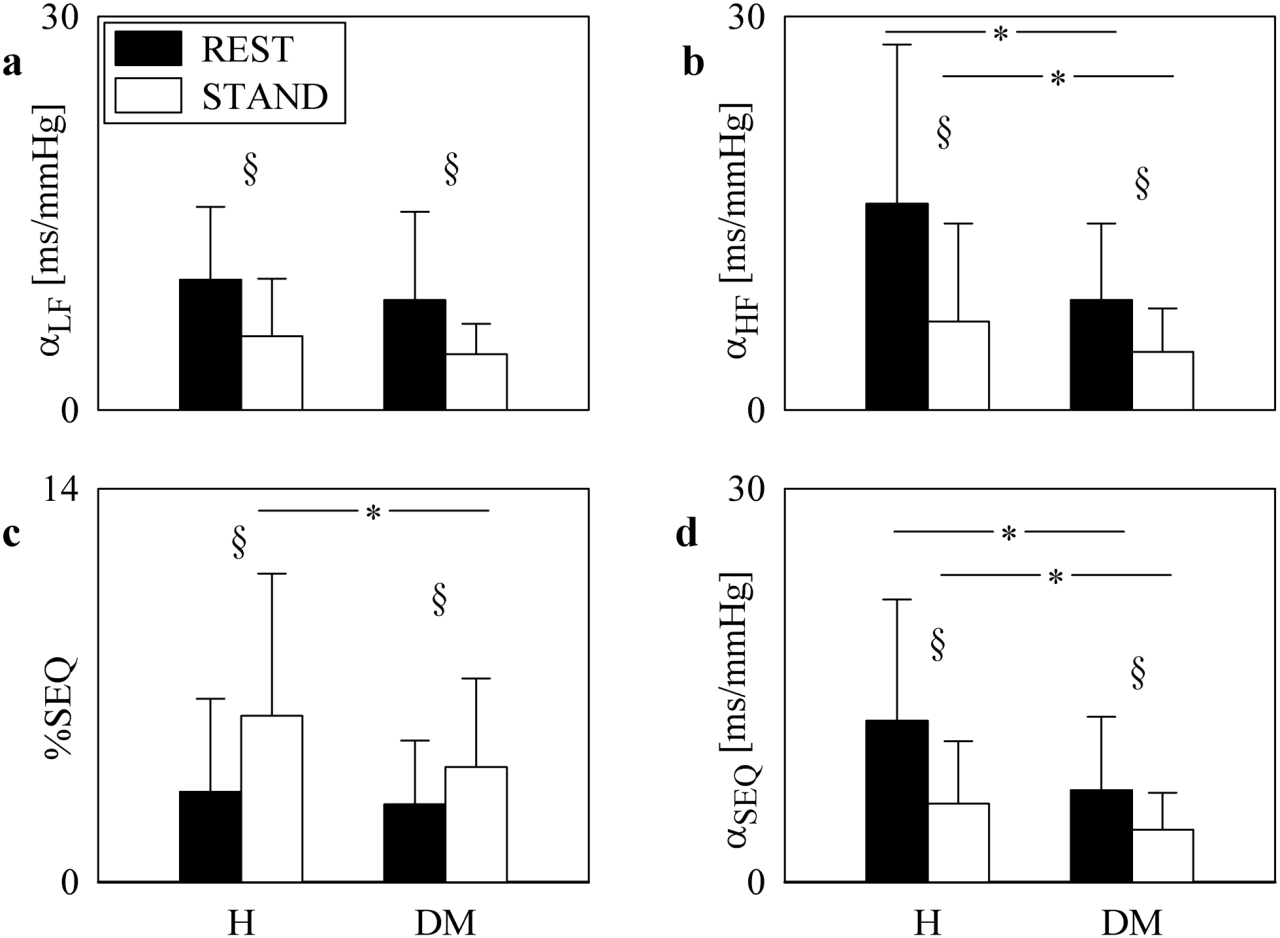
Time and frequency domain BRS parameters. The grouped bargraphs show the BRS estimates as a function of the group (i.e. H and DM individuals) at REST (black bars) and during STAND (white bars). The graphs are relevant to α_LF_ (a), α_HF_ (b), %SEQ (c), and α_SEQ_ (d). Values are given as mean plus standard deviation. The symbol * indicates differences between groups (i.e. H vs DM) with *p*<0.05, while the symbol § indicates differences between experimental conditions (i.e. REST vs STAND) with *p*<0.05.

The grouped bargraphs of Fig 3 show the BRS estimates (Fig 3a, 3b and 3d) as a function of the group (i.e. H and DM individuals) at REST (black bar) and during STAND (white bars). The percentage of baroreflex sequences, %SEQ, was reported as well (Fig 3c). Values are given as mean plus standard deviation. All BRS estimates decreased during STAND regardless of the group (i.e. in H or DM individuals). α_HF_ and α_SEQ_ was significantly smaller in DM patients than in H subjects regardless of the experimental conditions (Fig 3b and 3d). α_LF_ did not separate groups within the same experimental condition (Fig 3a). The efficacy of the orthostatic stimulus in both groups was confirmed by the increase of %SEQ during STAND regardless of the group (Fig 3c). %SEQ distinguished the groups only during STAND (Fig 3c). Considering separately up and down BRS sequences did not lead to results significantly different from those reported in Fig 3c and 3d.

## Discussion

The main findings of this study are as follows: i) the HF_HP_ power was lower in type 2 DM patients than in H subjects at REST, while the two groups had comparable HF_HP_ power during STAND; ii) the LF_SAP_ power was similar in type 2 DM patients and H subjects at REST and increased during STAND in both groups; iii) α_HF_ and α_SEQ_ in type 2 DM group was smaller than in H subjects at REST and both BRSs decreased during STAND.

### Novelty of the selected type-2 DM group and of the protocol design

H and DM groups were similar in terms of age and antropometric characteristics. As expected, they were different in relation to glycated hemoglobin (HbA1c), fasting plasma glucose, insulin and triglycerides [28,29]. The main elements of novelty of the type 2 DM group are: i) it is composed by non-smokers individuals that never smoked in their life; ii) it is composed by type 2 DM patients who were not affected by CAN and are asymptomatic for any type of neuropathy. The absence of CAN was verified according to Ewing tests assessing the heart rate at REST, the response of heart rate to slow deep breathing test, to STAND and Valsalva maneuver, the orthostatic hypotension in reaction to STAND [18]. The absence of peripheral neuropathy and other type of neuropathies was checked by evaluating the sensitivity to 10.0 g Semmes-Weinstein monofilament and by clinical anamnesis.

This special subgroup of type 2 DM patients was chosen because several studies observed that smoking affected the magnitude of heart rate variability and BRS [30], and it was shown that smoking negatively influences the autonomic control. It is well-known that individuals with CAN exhibited lower BRS [3,31]. This impairment was attributed to the functional or structural impairment of nerve fibers of the autonomic nervous system innervating the heart and blood vessels [32]. In addition, the presence of other neuropathies, e.g. peripheral neuropathy, was associated with a reduced BRS [8]. The selected population would allow us to assess the effect of type 2 DM on the autonomic function and baroreflex control of heart rate *per se* in absence of the confounding factors such as smoking and manifest neuropathies.

The element of originality of the experimental protocol lies in the exploitation of an active postural maneuver (i.e. STAND), being an important daily life stressor of the cardiovascular control [16,17] and easily applicable at the bedside in clinics. This maneuver has been used to probe baroreflex control of heart rate and challenge cardiovascular homeostasis [16,33,34]. Indeed, this maneuver causes a reduction of the venous return triggering an increase of heart rate and peripheral vascular resistance to maintain suitable values of arterial blood pressure [35]. This response is mediated by a cardiac vagal withdrawal and vascular sympathetic activation leading to a decrease of the HF_HP_ power and an increase of the LF_SAP_ one in H population [14,15]. These expected modification of the HF_HP_ and LF_SAP_ powers in response to STAND in H subjects were confirmed in present study (Fig 1e and 1f), thus remarking the suitability of this stimulus to probe autonomic control. In addition, STAND elicited between-group differences. Indeed, while the HF_HP_ power significantly decreased in response to STAND in H subjects, the decline was not visible in the DM group.

### Time and frequency domain parameters in the type-2 DM group

The DM group featured a reduced μ_HP_ compared to the H subjects but preserved the tachycardic response to STAND. In addition, the DM group was characterized by higher values of μ_SAP_ due to the presence of 15 subjects (44.12%) with arterial hypertension controlled by medication (Tab.1). The HF_HP_ power in the DM group was smaller than that in H subjects at REST and it was not significantly modified by STAND. Conversely, the LF_SAP_ power in the DM group was similar in H subjects at REST and it was significantly increased by STAND in both groups. These data allow us to conclude that the vagal control directed to the heart, as assessed by HF_HP_, is impaired in DM patients, while the sympathetic control directed to the vessels, as estimated by LF_SAP_, is preserved. This finding is in agreement with the original observation reported in [36] showing that individuals with type 2 DM feature first a vagal nerve injury and, later, a damage of the sympathetic fibers. Other study evaluating a DM population found a reduced LF_SAP_ power [6], but, at difference with the present study, their DM group included individuals with CAN. Therefore, we conclude that the presence of CAN or other type of neuropathies might be responsible for the additional impairment of the sympathetic control to the vessels, while the vagal impairment is a primary result of type 2 DM. It is worth noting that similar results were found in individuals with type 1 DM [37].

### Baroreflex control of heart rate in type-2 DM group

α_HF_ and α_SEQ_ were significantly smaller in DM patients than in H subjects. This finding reveals an impairment of baroreflex control of heart rate in DM group. Therefore, it seems that α_HF_ and α_SEQ_ are sensitive enough to detect the baroreflex dysfunction in individuals with type 2 DM without CAN and asymptomatic for any other type of neuropathy. This result could not be achieved using α_LF_, possibly due to a weaker statistical power of α_LF_ compared to α_HF_ and α_SEQ_. It is remarkable that our DM group preserved the ability to reduce BRS during STAND. Since in H subjects a decrease of BRS in response to STAND is expected [38] and it is confirmed by all BRS estimates computed in this study (Fig 3a, 3b and 3d), we suggest that the response of cardiac baroreflex was maintained in DM patients. Since an impaired cardio-vagal and vasomotor response to baroreceptor stimulation was found in subjects with type 2 DM with more than two abnormal Ewing tests [7], we concluded that CAN and/or other neuropathies might be responsible for the impairment of the baroreflex response to STAND, while type 2 DM *per se* might leave this ability unharmed.

### Limitations of the study and future developments

One of the possible confounding factors of the study is that it is controlled in terms of CAN and other neuropathies (i.e. their effect on the results are excluded) but it is not controlled for other possible co-morbidities of type 2 DM. One of the co-morbidity is hypertension. However, when analysis was carried out over the non-hypertensive group results were not significantly different from those here reported, thus suggesting the that presence of a fraction of hypertensive patients in the DM group did not influence the conclusions of the study. We advocate studies specifically designed to rule out the impact of hypertension and hypertensive medications in a population of type 2 DM in absence of CAN and/or other neuropathies. Future studies should assess the potential effect of countermeasures in controlling and even reversing the vagal autonomic dysfunction and to check whether the worsening effect of incoming neuropathy over the autonomic function could depend on the type of neuropathy.

## Conclusions

Individuals with type 2 DM without CAN and asymptomatic for any other type of neuropathy featured an impairment of vagal regulation and the baroreflex control of heart rate, maintained intact the sympathetic control directed to vessels and preserved the capability of cardiac baroreflex and sympathetic control to react to postural challenge. The autonomic control profile of our DM patients is not favorable because a lower BRS reduces the ability of cardiac baroreflex to deal with large variations in arterial blood pressure and the reduced vagal modulation might exert an insufficient restraint against sympathetic hyper-tone, thus exposing type 2 DM individuals without CAN and asymptomatic for any other type of neuropathy to risky situations such as stroke and cardiac arrhythmias. We conclude that analysis of spontaneous fluctuations of HP and SAP provide indexes sensitive enough to detect cardiovascular dysfunction in type 2 DM patients without CAN and asymptomatic for any other type of neuropathy and to typify it. In addition, data suggest that type 2 DM affected *per se* baroreflex control of heart rate through an impairment of vagal control.
