# Correction: Cardiovascular Variability Analysis and Baroreflex Estimation in Patients with Type 2 Diabetes in Absence of Any Manifest Neuropathy

**DOI:** 10.1371/journal.pone.0174492

**Published:** 2017-03-16

**Authors:** Sílvia Cristina Garcia de Moura-Tonello, Alberto Porta, Andrea Marchi, Alessandra de Almeida Fagundes, Cristina de Oliveira Francisco, Patrícia Rehder-Santos, Juliana Cristina Milan-Mattos, Rodrigo Polaquini Simões, Mariana de Oliveira Gois, Aparecida Maria Catai

The Data Availability statement for this paper is incorrect. The correct statement is: All relevant data are available on the Dryad repository (http://dx.doi.org/10.5061/dryad.5d8jv).
